# Supplementary material for: MetaRibo-Seq measures translation in microbiomes
Source: Nat Commun. 2020 Jun 29;11:3268. doi: 10.1038/s41467-020-17081-z (PMC7324362; doi:10.1038/s41467-020-17081-z)
Supplement: Supplementary file 10 — Supplementary Data 7 [file 41467_2020_17081_MOESM10_ESM.zip › File2/Confidence_High_Taxonomy/233158_out.krona.html]

Javascript must be enabled to view this page.

members
magnitude
magnitudeUnassigned
count
unassigned
taxon
rank

233158\_out

2

2
2759
superkingdom

2
4751
kingdom

451864
2
subkingdom

2
4890
phylum

subphylum
147538
2

class
147548
2

order
5178
2

genus
453209
2

species
2
1849047

SRS078419\_contig\_number\_21397SRS104311\_contig\_number\_contig-100\_3102.240645
